# ANXA6/TRPV2 axis promotes lymphatic metastasis in head and neck squamous cell carcinoma by inducing autophagy

**DOI:** 10.1186/s40164-023-00406-1

**Published:** 2023-05-03

**Authors:** Min Wang, Min Pan, Yanshi Li, Tao Lu, Zhihai Wang, Chuan Liu, Guohua Hu

**Affiliations:** Department of Otorhinolaryngology, The First Affiliated Hospital of Chongqing Medical University, Chongqing, 400016 China

**Keywords:** ANXA6, TRPV2, Head and neck squamous cell carcinoma, Autophagy, Lymphatic metastasis

## Abstract

**Background:**

Head and neck squamous cell carcinoma (HNSCC) is highly aggressive with a significant tropism of lymph nodes, which restricts treatment options and negatively impacts patient outcomes. Although progress has been made in understanding the molecular mechanisms underlying lymphatic metastasis (LM), these mechanisms remain elusive. ANXA6 is a scaffold protein that participates in tumor pathogenesis and autophagy regulation; however, how ANXA6 affects autophagy and LM in HNSCC cells remains unknown.

**Methods:**

RNA sequencing was performed on HNSCC clinical specimens with or without metastasis as well as on The Cancer Genome Atlas dataset to investigate ANXA6 expression and survival. Both in vitro and in vivo studies were performed to investigate the role of ANXA6 in the regulation of LM in HNSCC. The molecular mechanism by which ANXA6 interacts with TRPV2 was examined at the molecular level.

**Results:**

ANXA6 expression was significantly upregulated in HNSCC patients with LM and higher expression was associated with poor prognosis. ANXA6 overexpression promoted the proliferation and mobility of FaDu and SCC15 cells in vitro; however, ANXA6 knockdown retarded LM in HNSCC in vivo. ANXA6 induced autophagy by inhibiting the AKT/mTOR signaling pathway in HNSCC, thereby regulating the metastatic capability of the disease. Furthermore, ANXA6 expression positively correlated with TRPV2 expression both in vitro and in vivo. Lastly, TRPV2 inhibition reversed ANXA6-induced autophagy and LM.

**Conclusions:**

These results indicate that the ANXA6/TRPV2 axis facilitates LM in HNSCC by stimulating autophagy. This study provides a theoretical basis for investigating the ANXA6/TRPV2 axis as a potential target for the treatment of HNSCC, as well as a biomarker for predicting LM.

**Supplementary Information:**

The online version contains supplementary material available at 10.1186/s40164-023-00406-1.

## Background

Head and neck squamous cell carcinoma (HNSCC) is the most common type of head and neck cancer, and originates from the mucosal epithelium of the mouth, pharynx, and larynx. It is the sixth leading cause of cancer-related death worldwide [1–4]. Currently, surgery, adjuvant radiotherapy, and chemotherapy are the primary treatment strategies for HNSCC. However, the complex anatomical structure and concealability of the disease as well as the high demand for functional preservation make the choice of surgical approach more challenging, resulting in a low surgical cure rate and poor postoperative prognosis, with approximately 60% survival at 5 years [2, 5]. Lymphatic metastasis (LM) and degree of primary tumor invasion are the main factors affecting the prognosis of HNSCC [6]. In previous studies, LM has been shown to be an independent prognostic factor for HNSCC [7, 8]. Therefore, detailed molecular characterization of lymphatic metastases may help identify targeted therapies and enable prolonged survival. Much progress has recently been made in understanding the molecular mechanisms underlying LM; however, the underlying mechanisms are not fully understood.

ANXA6 is a calcium-dependent membrane-bound annexin involved in membrane trafficking, membrane and cytoskeletal organization, cholesterol homeostasis, and signal transduction [9]. It plays an essential role in several tumor types, and its expression in tumors is primarily determined by the type and degree of malignancy. This gene has been reported to inhibit tumor spread and proliferation in gastric, breast, and pancreatic cancer [10–12]. Noreen et al. identified ANXA6 as an oncogene that promotes tumor progression [13]. However, there is no evidence that ANXA6 is involved in HNSCC development. The detailed role of ANXA6 in LM requires further investigation.

Tumor cells can invade and metastasize, which is not only essential for tumor progression but also the primary cause of death for patients [14]. In clinical practice, most patients with HNSCC develop LM during their first visit, resulting in a significantly worse survival prognosis [15]. In LM, tumor cells migrate through the lymphatic vessels to drain the tumor-draining lymph nodes. It is also possible for LM to cause hematogenous dissemination throughout the body [16, 17]. The mechanisms of autophagy are conserved homeostasis mechanisms that degrade proteins, organelles, and other structures [14, 18]. Autophagy promotes tumor proliferation, invasion, and migration [14, 19, 20]. The AKT/mTOR signaling pathway is the main pathway regulating autophagy, which determines the survival and death of tumor cells and plays a crucial role in tumorigenesis [21]. Recently, Sun et al. reported that ANXA6 expression inhibits the AKT/mTOR signaling pathway to activate autophagy and affect cervical cancer progression [22]. However, whether ANXA6 regulates HNSCC autophagy via the AKT/mTOR pathway to promote LM remains unclear.

Bioinformatics analysis of cancer patient data from public databases, such as The Cancer Genome Atlas (TCGA), has provided bioinformatics data on many common tumors [23]. Thus, we analyzed the TCGA data for head and neck tumors. As a member of the transient receptor potential (TRP) family, TRPV2 is highly correlated with ANXA6. It is a Ca^2+^ osmotic ion channel activated by heat, osmotic pressure changes, and membrane stretching, and is involved in tumorigenesis and metastasis [24–26]. However, the involvement of TRPV2 in autophagy and LM of HNSCC remains unclear.

In this study, ANXA6 was screened by transcriptome sequencing of HNSCC tissues with and without LM. Through in vitro and in vivo experiments, ANXA6 was found to inhibit mTOR phosphorylation by regulating TRPV2 and inducing autophagy in HNSCC cells. These findings provide important information for the identification of biomarkers and new therapeutic targets for LM in HNSCC.

## Materials and methods

### HNSCC clinical specimens and RNA sequencing

We collected 20 pairs of HNSCC tissue samples with LM, 20 pairs of HNSCC tissue samples without LM (NLM), and their corresponding normal adjacent tissues (NAT) from the First Affiliated Hospital of Chongqing Medical University between 2012 and 2022. The inclusion criterion was HNSCC resection without chemoradiotherapy. Three cases of HNSCC primary lesions with LM and three cases without LM were selected for transcriptome sequencing. Specific methods and data analysis are described in detail in our previous study [27]. The remaining fresh tissues were stored at − 80 °C for subsequent experiments. Paraffin-embedded sections were obtained from 78 patients with HNSCC. All patients underwent surgical treatment between 2012 and 2019. This study was approved by the Ethics Committee of First Affiliated Hospital of Chongqing Medical University. Informed consent was obtained from all patients.

### Cell lines and transfection

Human pharyngeal squamous cell carcinoma cell (FaDu cell) and human tongue squamous cell carcinoma cell (SCC15 cell) were purchased from the Cell Bank of the Chinese Academy of Sciences (Shanghai, China). FaDu cells were cultured in minimal essential medium containing 10% fetal bovine serum (FBS) and 1% penicillin/streptomycin (PS) (Gibco, Billings, MT, USA) at 37 °C with 5% CO_2_. SCC15 cells were cultured in high glucose Dulbecco’s modified Eagle medium (DMEM; Gibco).

ANXA6 knockdown (KD) in HNSCC cell lines (FaDu and SCC15) was performed using small interfering RNA (siRNA) (GenePharma, China). The detailed method was as follows: HNSCC cells were cultured in 6-well plates to approximately 60–70% confluence. The siRNA (Additional file 1: Table S1) and transfection reagent (Lipofectamine RNAiMAX; Invitrogen, Waltham, MA, USA) were added at a ratio of 3:6, respectively, incubated with Opti-MEM (Gibco) for 6–8 h, and then changed to medium containing FBS and PS. After 48–72 h, RNA and proteins were extracted for quantitative real-time PCR (qRT-PCR) and western blot analyses, respectively, to verify transfection efficiency.

Stably expressing HNSCC cell lines were constructed using an overexpression vector and lentiviral short hairpin RNA (shRNA) customized according to the siRNA transfection efficiency (sequence GGGACUUUGAGAAGCUAAUTT, AUUAGCUUCUCAAAGUCCCTT; GenePharma, China). The overexpression vector was GV208, and the sequence of the vector elements was UBI-MCS-Firefly-Luciferase-IRES-Puromycin. The shRNA vector was GV344, and the sequence of the carrier elements was HU6-MCS-Ubiquitin-firefly-Luciferase-IRES-Puromycin (Genechem, Shanghai, China). The specific steps were as follows: When HNSCC cells were approximately 50% confluent, the corresponding proportions of lentivirus and infection reagent (Genechem, Shanghai, China) were added according to the multiplicity of infection (MOI), and the culture medium was changed to normal medium for 16–18 h. When the cell density reached 80–90%, the medium containing 2 µg/ml puromycin was replaced until the wild-type cells died. The medium containing 1 µg/ml puromycin was maintained for 2 weeks to obtain stably expressing HNSCC cell lines for subsequent experiments.

### qRT-PCR

qRT-PCR was performed as described previously [28]. Briefly, we isolated RNA from HNSCC patients with or without LM, as well as from a cell line, using the E.Z.N.A. Total RNA Kit I (Omega Bio-Tek, Norcross, USA). The PrimeScript RT reagent kit (Takara, Dalian, China) was used to reverse transcribe RNA into cDNA. Next, qRT-PCR was performed using SYBR Green (Takara, Dalian, China) per the manufacturer’s instructions. GAPDH was used as an internal control to calculate relative mRNA expression. The detailed primer sequences are provided in Additional file 1: Table S1.

### Western blot analysis

Western blotting was performed as described previously [29]. Briefly, proteins extracted from tumor samples of HNSCC, FaDu, and SCC15 cells were separated using SDS-PAGE and then transferred onto PVDF membranes (Beyotime, Shanghai, China). As listed in Additional file 1: Table S2, the membranes were incubated overnight at 4 °C with primary antibodies. After washing, membranes were incubated with horseradish peroxidase-conjugated secondary antibodies. GAPDH was used as a normalization reference to analyze the bands using ImageJ.

### Immunohistochemistry and immunofluorescence staining

Immunohistochemistry (IHC) staining was performed as previously described [28]. Briefly, IHC was performed on 78 paraffin-embedded HNSCC tissues (55 with LM and 23 without LM). Slides were incubated with diluted primary antibodies (listed in Additional file 1: Table S2) after antigen retrieval and peroxidase blocking. Next, the slides were treated with a boost IHC detection reagent (ZSGB-BIO, Guangzhou, China). After staining with DAB, slides were counterstained with hematoxylin. The immunohistochemistry results were analyzed separately by two experienced pathologists and scored based on both the staining intensity and the percentage of tumor cells with an unequivocal positive response. The negative control consisted of slides incubated with phosphate-buffered saline (PBS) instead of primary antibody. The intensities of AXNA6 and TRPV2 were calculated based on the staining results by multiplying the signaling intensity score by the staining distribution score, as previously reported [28].

For immunofluorescence (IF) staining, FaDu and SCC15 cells were fixed in 4% paraformaldehyde for 20 min, permeabilized with 0.1% Triton X-100 for 15 min, and blocked with goat serum at room temperature for 30 min. Afterward, the cells were incubated with primary antibodies (listed in Additional file 1: Table S2) overnight at 4 °C. Fluorescence-labeled secondary antibodies were used to incubate the cells on the following day. We stained nuclear DNA with DAPI (C1006; Beyotime, Shanghai, China) and used a Nikon confocal system for image capture.

### Cell proliferation assay

Cell Counting Kit-8 (CCK-8; Beyotime, Shanghai, China) and EdU (RIBOBIO, Guangzhou, China) assays were used to detect the proliferation of HNSCC cells. Briefly, transfected HNSCC cells were cultured in 96-well plates and incubated for 1 h at 37 °C with the addition of a medium containing 10% CCK-8 solution. Finally, the absorbance values were measured continuously at 450 nm for 4 days using a microplate reader (iMark Microplate Absorbance Reader; Bio-Rad Laboratories, Hercules, CA, USA). Similarly, the transfected HNSCC cells (8 × 10^3^ cells/well) were seeded into 96-well plates. After 36 h, experiments were performed according to the manufacturer’s instructions for the EdU detection kit.

### Cell migration and invasion assays

Wound healing and Transwell assays were used to observe the migration and invasion abilities of HNSCC cells. The steps are described in detail in our previous study [30].

### Cell cycle and apoptosis assays

For the cell cycle assay, stably transfected lentivirus cells were cultured to approximately 80% confluence, digested with trypsin, washed with PBS, resuspended with 100 µl PBS, and fixed in 900 µl 70% ice-cold ethanol at 4 °C overnight. The cell cycle was determined after treatment with RNase A and propidium iodide using flow cytometry (Avidity Biosciences, San Diego, CA, USA).

For the cell apoptosis assay, cells (sh-NC-FaDu, sh-ANXA6-FaDu, sh-NC-SCC15, sh-ANXA6-SCC15, vector-FaDu, OE-ANXA6-FaDu, vector-SCC15, and OE-ANXA6-SCC15) were cultured in 6-well plates until overgrowth. The floating cells were collected and digested with EDTA-free trypsin, washed with PBS, centrifuged again, and resuspended in 500 µl PBS. Finally, the cells were labeled with a binding buffer containing Annexin V-FITC/PI, and the samples were analyzed using flow cytometry (Avidity Biosciences).

### LM model of HNSCC in vivo

An animal model of HNSCC LM was established by injecting 5 × 10^6^ cells (sh-ANXA6 and sh-NC groups) into the footpads of 5–6 week-old nude mice (Beijing Hufukang Biotechnology Co., Ltd., China). The body weights and tumors of the mice were observed and recorded once per week. After 5 weeks, small animal imaging was performed (IVIS; Berthold Technologies, Baden Wurttemberg, Germany), and mice were sacrificed to obtain primary tumors and corresponding ipsilateral lymph nodes. Parts of the lymph node tissues were used as pathological paraffin sections for hematoxylin and eosin (H&E) staining. The study protocol was approved by the Animal Care and Treatment Committee of Chongqing Medical University.

### Transmission electron microscopy

HNSCC cells (vector-FaDu, OE-ANXA6-FaDu, vector-SCC15, and OE-ANXA6-SCC15) were cultured at 5 × 10^6^ and then digested with 0.1% trypsin. The fixation solution was then added and the sections were embedded. Finally, transmission electron microscopy (Hitachi, Tokyo, Japan) was used to scan and capture representative fields.

### Statistical analysis

All values are expressed as mean ± standard deviation (SD). The Pearson χ^2^ test was used to determine the correlation between ANXA6 expression and clinicopathological characteristics of HNSCC patients, whereas Kaplan–Meier survival curves were used to assess the association between ANXA6 expression and 5-year all-cause mortality in patients with HNSCC. Student’s t-tests were used to compare differences between two groups, whereas one-way ANOVAs followed by Tukey’s post-hoc tests were used to compare multiple groups. Statistical analysis was conducted using GraphPad Prism software (version 9.3; GraphPad Software, San Diego, CA, USA), and a *P*-value < 0.05 was considered significant. Each experiment was independently repeated at least three times.

## Results

### ANXA6 is highly expressed in HNSCC with LM and affects survival

**Fig. 1:**
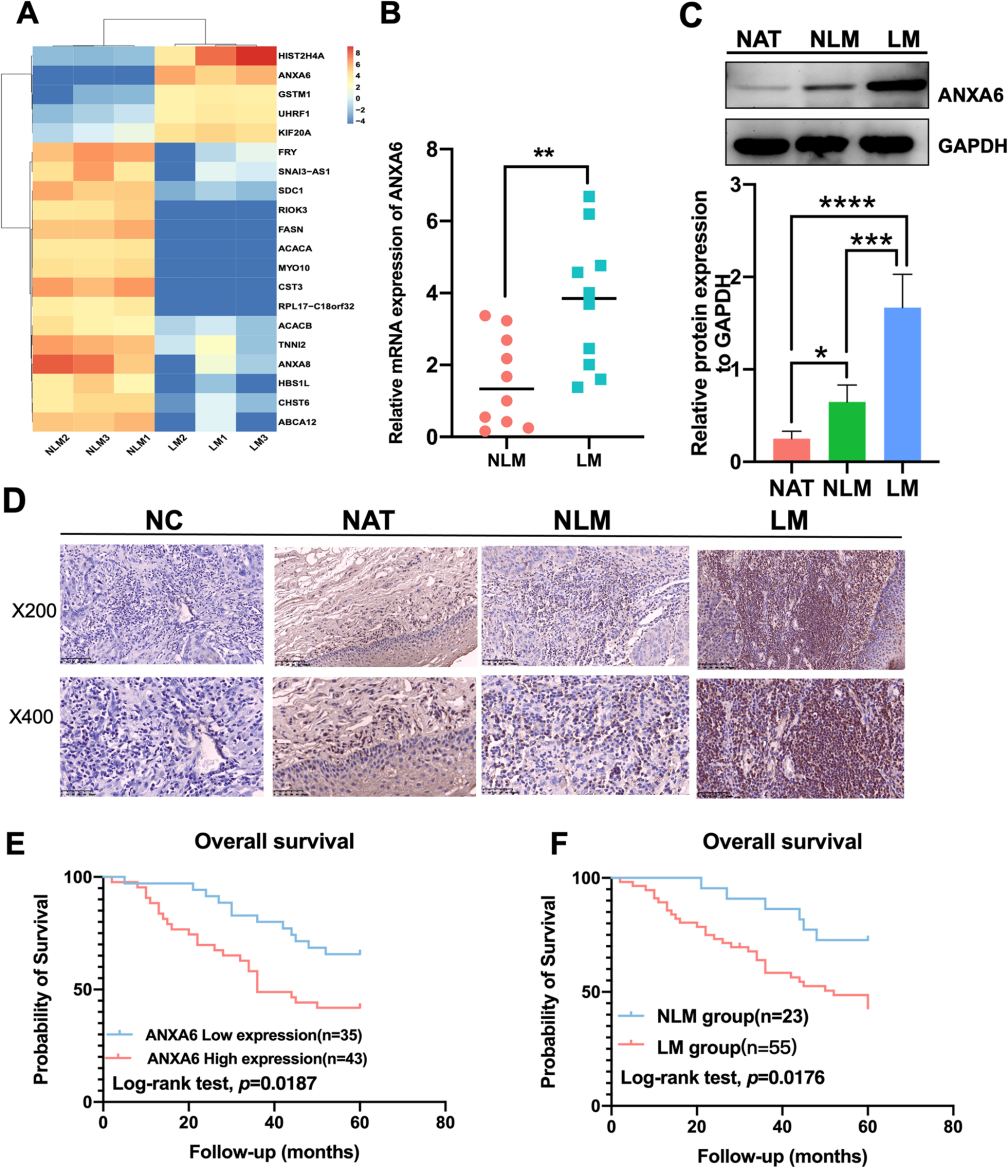
The expression of ANXA6 in patients with HNSCC. **A** Heat map shows the mRNA expression levels of differentially expressed genes in three pairs of HNSCC tissues. Red color indicates upregulated, and blue color indicates downregulated. **B** Relative mRNA expression levels of ANXA6 in HNSCC tissues with and without LM (n = 10). **C** The protein expression levels of ANXA6 in HNSCC tissues with and without LM and NAT (n = 6). **D** Representative images of IHC staining of ANXA6 in HNSCC tissues with and without LM, HNSCC tissue stained with PBS instead of primary antibody was as negative control (NC). **E** Comparisons of overall survival between low ANXA6 expression group and high ANXA6 expression group. **F** Comparisons of overall survival between LM group and NLM group. *LM* lymphatic metastasis, *NLM* non-lymphatic metastasis, *NAT* normal adjacent tissues. Data are presented as mean ± SD. *p < 0.05, **p < 0.01, ***p < 0.001, ****p < 0.0001 ; compared with the indicated group

Six HNSCC primary tumors (three with LM and three without LM) were analyzed using whole-transcriptome sequencing, and the top 20 differential genes were identified using heat maps. ANXA6 was found to be one of the most differentially expressed genes (Fig. 1A). We then verified ANXA6 expression in clinical HNSCC specimens. The results showed that ANXA6 mRNA levels were significantly higher in the LM group than in the NLM group (Fig. 1B). Consistent with the mRNA levels, western blotting confirmed higher expression of ANXA6 in the LM group (Fig. 1C). To further evaluate the expression of ANXA6, IHC staining was conducted on 78 HNSCC cases with complete clinical data and follow-up (Fig. 1D; Table 1), showing that it was significantly higher in the LM group compared with the NLM and NAT groups. In addition, higher ANXA6 expression was associated with pathological N stage and LM. More importantly, the Kaplan–Meier curve indicated that both high ANXA6 expression and LM predicted poor outcomes in the 78 HNSCC cases with follow-up (Fig. 1E, F). Taken together, these results indicate that high ANXA6 expression is closely associated with poor clinical outcomes in HNSCC and is possibly involved in LM.

**Table 1:** Expression and clinicopathological characteristics of ANXA6 in HNSCC

	Total	ANXA6 expression		*χ*^2^ value	*P* value
		High	Low		
Age (Y)				1.327	0.249
≥ 60	48	24	24		
< 60	30	19	11		
Gender				1.245	0.265
Male	77	43	34		
Female	1	0	1		
Pathological T stage				2.165	0.539
T1	4	3	1		
T2	13	9	4		
T3	36	18	18		
T4	25	13	12		
Pathological N stage				28.50	< 0.0001
N0	23	2	21		
N1	11	8	3		
N2	39	29	10		
N3	5	4	1		
Lymph node metastasis				28.43	< 0.0001
Yes	55	41	14		
No	23	2	21		
Extranodal extension				3.213	0.073
Yes	16	12	4		
No	62	31	31		
Tumor differentiation				1.007	0.605
Well	15	10	5		
Moderate	48	25	23		
Poor	15	8	7		

*ANXA6* annexin A6, *HNSCC* head and neck squamous cell carcinoma, *Y* year. *T* tumor, *N* node

### ANXA6 impacts HNSCC cells proliferation, apoptosis, and mobility in vitro

**Fig. 2:**
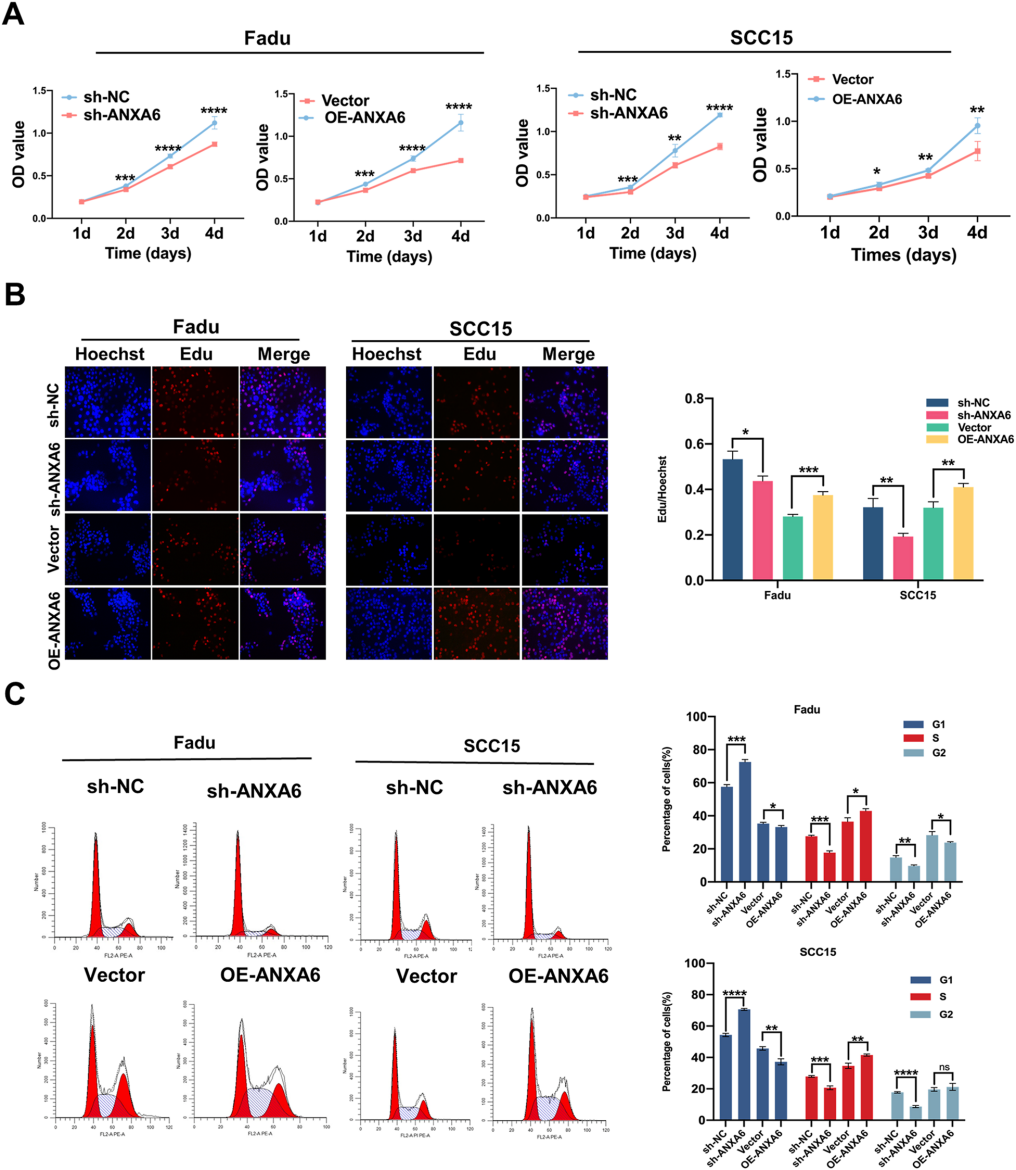
ANXA6 influences HNSCC cells proliferation in vitro. **A** CCK8 assays were used to assess the viability of FaDu and SCC15 cell lines after knockdown or overexpression of ANXA6. **B** EdU assays were used to assess the cell proliferation ability of FaDu and SCC15 cell lines after knockdown or overexpression of ANXA6. Histogram showing the proliferation rates of transfected cells in the corresponding groups. **C** Flow cytometry shows the effect of knockdown or overexpression of ANXA6 on cell cycle progression and cell proliferation in FaDu and SCC15 cell lines. Data are presented as mean ± SD. *p < 0.05, **p < 0.01, ***p < 0.001, ****p < 0.0001; compared with the indicated group

**Fig. 3:**
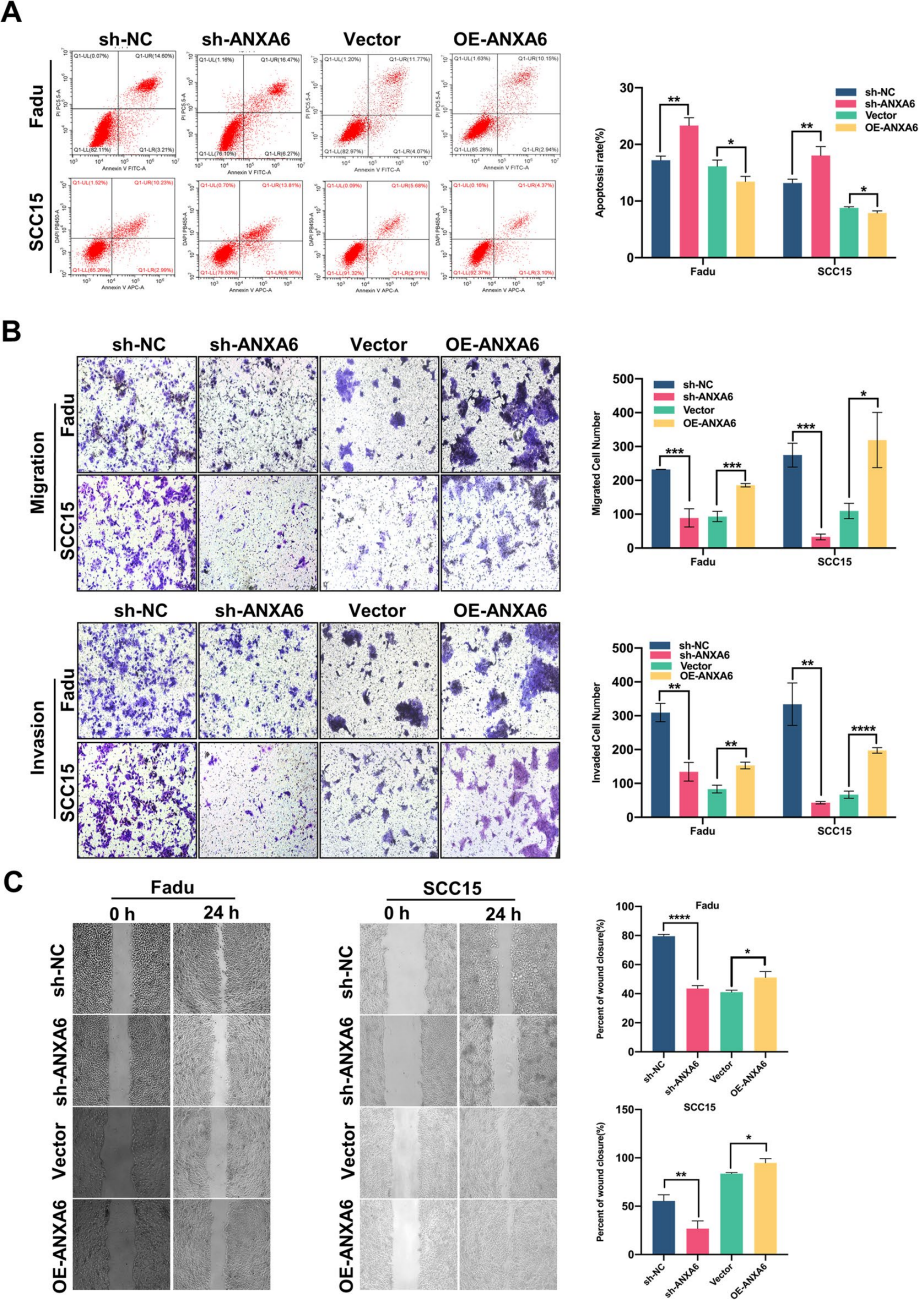
ANXA6 influences HNSCC cells apoptosis and mobility in vitro. **A** Flow cytometric analysis of apoptotic cells after knockdown or overexpression of ANXA6. Apoptotic rates were analyzed by fluorescence-activated cell sorting following treatment with virosecurinine. **B**, **C**. Transwell assay (**B**) and wound healing assay (**C**) were used to assess the migration and invasion abilities in FaDu and SCC15 cell lines after knockdown or overexpression of ANXA6. Histogram showing the numbers of migrated and invaded HNSCC cells. Data are presented as mean ± SD. *p < 0.05, **p < 0.01, ***p < 0.001, ****p < 0.0001; compared with the indicated group

Next, using the two HNSCC cell lines FaDu and SCC15, ANXA6 was overexpressed or KD via lentivirus transfection, which was verified by qRT-PCR and western blotting (Additional file 1: Fig. S1A–C). ANXA6 KD inhibited the proliferation of FaDu and SCC15 cells, and the proportion of S-phase cells decreased compared to that in the control group, whereas overexpression of ANXA6 produced opposite results (Fig. 2A–C). Additionally, apoptosis increased with ANXA6 KD. In contrast, ANXA6 overexpression significantly decreased apoptosis (Fig. 3A). Moreover, ANXA6 KD inhibited the migration and invasion of FaDu and SCC15 cells (Fig. 3B). These results were confirmed by a wound healing experiment (Fig. 3C). Collectively, these results indicate that ANXA6 participates in the apoptosis, proliferation, and mobility of HNSCC cells.

### ANXA6 KD inhibits tumor growth and LM in vivo

**Fig. 4:**
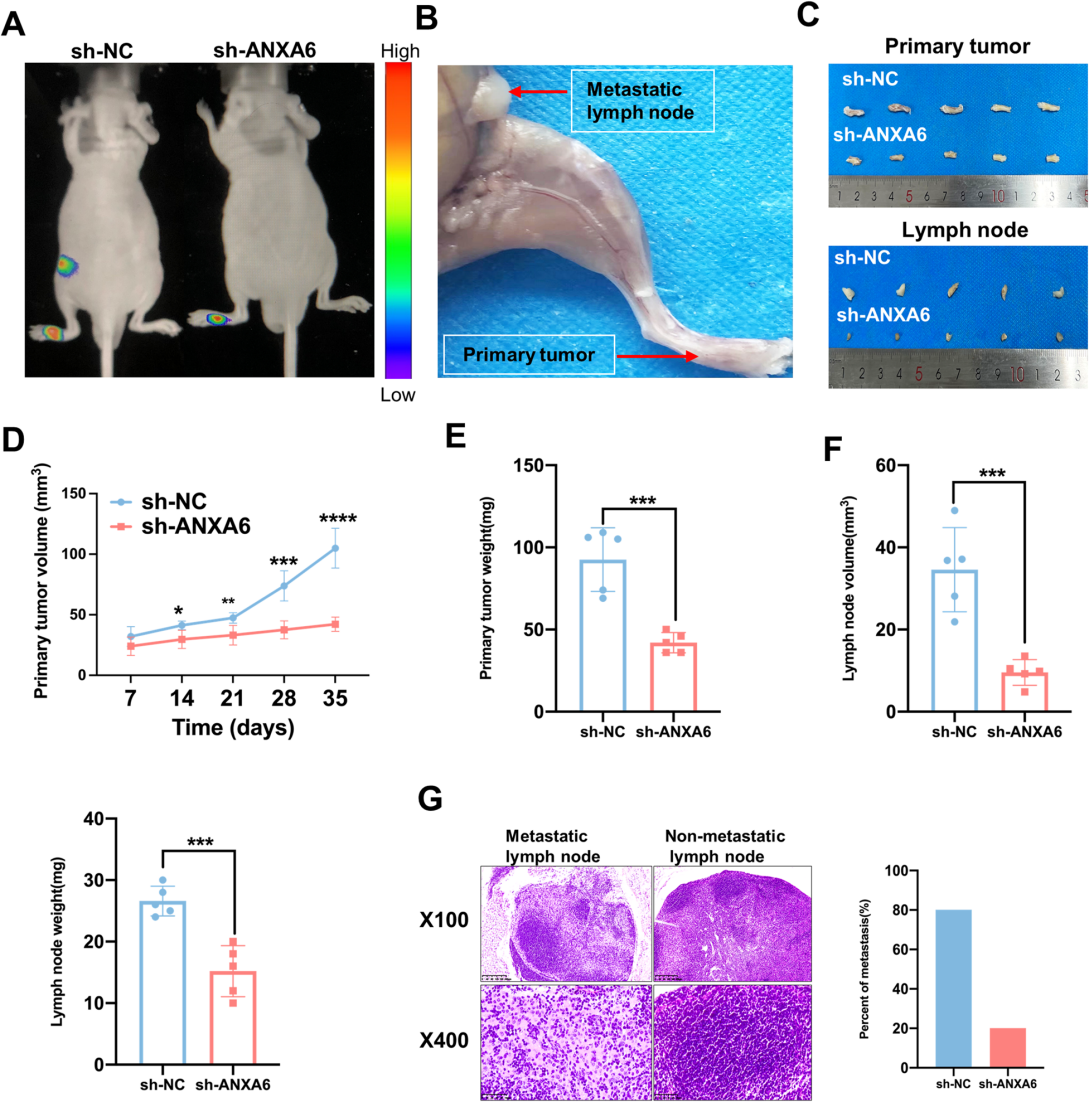
ANXA6 silencing can inhibit tumor growth and lymphatic metastasis in vivo. **A** Representative images of HNSCC lymphatic metastasis model (FaDu cells were injected into the foot pads of nude mice and observed for 35 days) for in vivo imaging and fluorescence representing tumorigenesis (n = 5, red color means strong fluorescence, purple means weak). **B** Representative images of lymphatic metastasis in nude mice. **C** Representative images of primary tumors and lymph nodes from nude mice (n = 5). **D**–**F**. Volume and weight of primary tumor and lymph nodes from nude mice. **G** Representative images of H&E staining of lymph node metastasis and percentage of metastasis (n = 5). Data are presented as mean ± SD. *p < 0.05, **p < 0.01, ***p < 0.001, ****p < 0.0001; compared with the indicated group

Based on the unexpected findings in vitro, we investigated the role of ANXA6 in regulating metastasis in vivo. A model of HNSCC LM was established by injecting lentivirus-transfected FaDu cells expressing ANXA6 KD (sh-ANXA6) or a negative control (sh-NC) into the foot pads of nude mice. Mice in the sh-ANXA6 group showed significantly lower LM fluorescence intensity after 35 days than mice in the sh-NC group (Fig. 4A). In addition, the primary tumor and lymph nodes were much smaller (Fig. 4B, C), and the volume and weight of the primary tumor and lymph nodes were less in the sh-ANXA6 group than in the sh-NC group (Fig. 4D–F). Finally, H&E staining revealed a lower rate of LM in the sh-ANXA6 group (Fig. 4G). Consistent with the in vitro experiments, ANXA6 KD inhibited tumor growth and metastasis in vivo.

### ANXA6 regulates metastatic ability of HNSCC by inducing autophagy

**Fig. 5:**
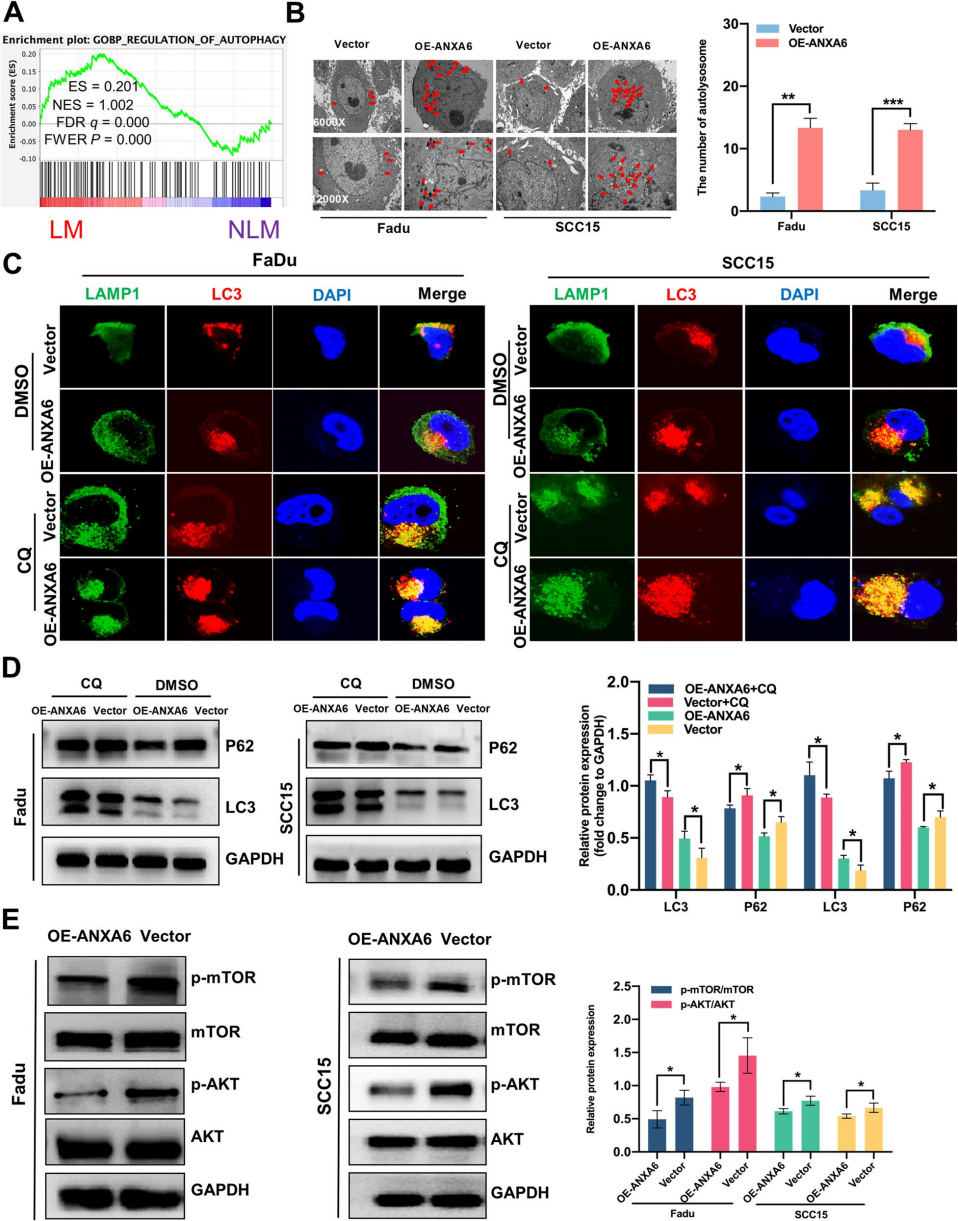
ANXA6 promotes autophagy by inhibiting the AKT/mTOR pathway in HNSCC cells. **A** The gene set enrichment analysis (GSEA) using transcriptome sequencing shows autophagy signaling pathway enriched in the HNSCC patients with lymphatic metastasis. **B** Representative transmission electron microscope (TEM) images of autophagosomes (red arrow) in FaDu and SCC15 cell lines after overexpression ANXA6. Magnification: ×6000 for low-power fields, ×12,000 for high-power fields. **C** Representative immunofluorescent images of the colocalization of LC3 (red) and LAMP1 (green) after overexpression ANXA6 in HNSCC cells treated with DMSO (10 μm/ml) or CQ (10 μm/ml) for 24 h. **D** The protein expression levels of autophagy-related markers, P62 and LC3 in HNSCC cells treated with DMSO (10 μm/ml) or CQ (10 μm/ml) for 24 h. **E** The protein expression levels of p-AKT, AKT, p-mTOR, and mTOR after overexpression of ANXA6 in HNSCC cells. Data are presented as mean ± SD. *p < 0.05, **p < 0.01, ***p < 0.001; compared with the indicated group

**Fig. 6:**
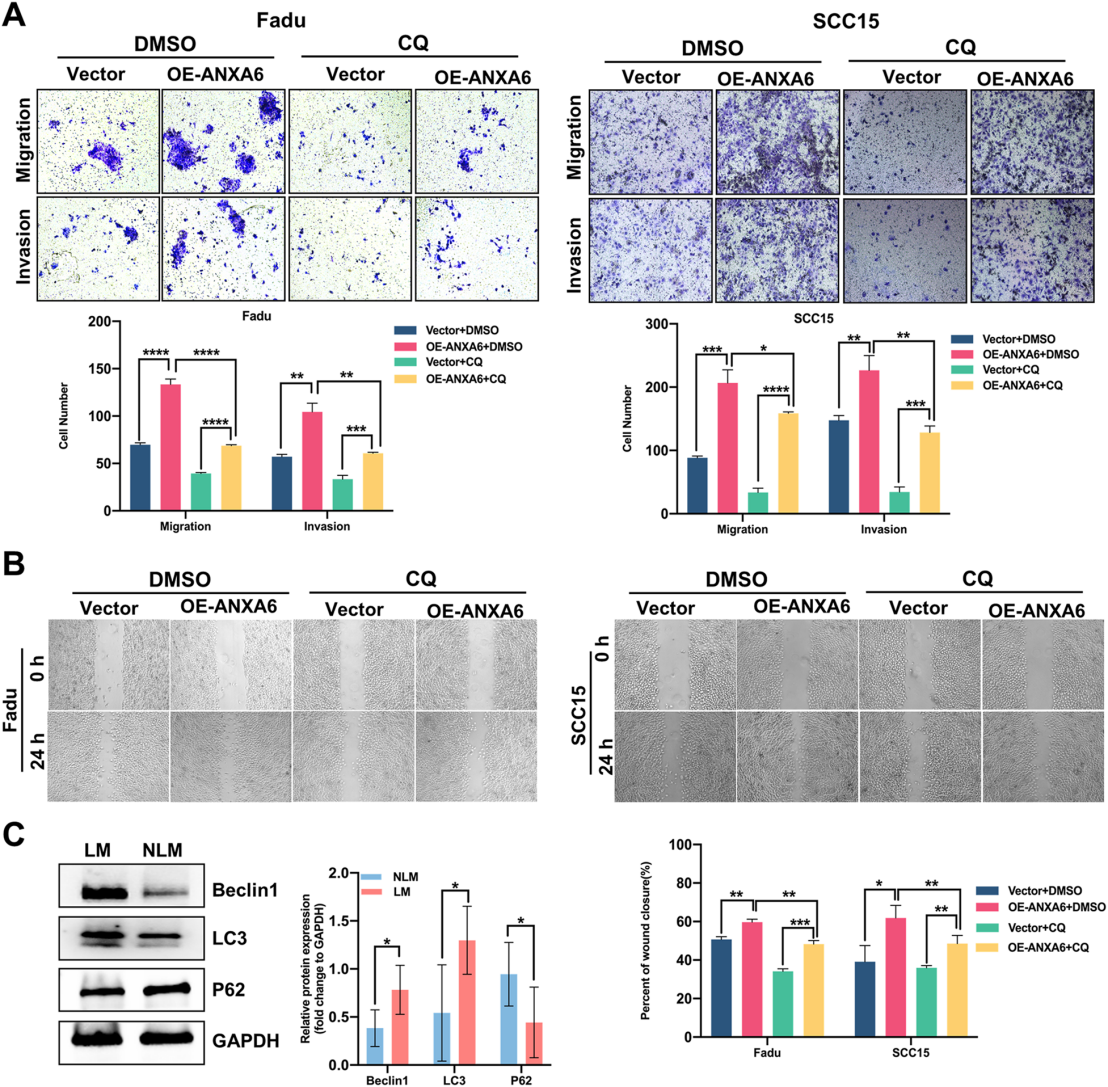
The effect of autophagy on HNSCC metastasis in vitro and in vivo. **A**, **B** Transwell assay (**A**) and wound healing assay (**B**) were used to assess the migration and invasion abilities in FaDu and SCC15 cell lines after overexpression of ANXA6 and dealing with DMSO (10 μm/ml) or CQ (10 μm/ml) for 24 h. **C** The protein expression levels of Beclin1, LC3, and P62 in HNSCC patients with and without lymphatic metastasis (n = 12). Data are presented as mean ± SD. *p < 0.05, **p < 0.01, ***p < 0.001, ****p < 0.0001; compared with the indicated group

Accumulating evidence has demonstrated that autophagy plays a vital role in the metastasis of various types of tumors [31, 32]. Therefore, we speculated that autophagy plays a critical role in HNSCC metastasis. Gene Set Enrichment Analysis (GSEA) indicated that autophagy favors HNSCC with LM (Fig. 5A). Autophagy involves autophagosome formation. Ultrastructural observations and immunofluorescence revealed that more autophagosomes and autophagic lysosomes were present in the ANXA6 overexpression group (OE-ANXA6) (Fig. 5B, C) and autophagy-related markers (Fig. 5D). We then preincubated ANXA6-overexpressed FaDu and SCC15 cells with chloroquine (CQ), an autophagy inhibitor. ANXA6 overexpression induced autophagy (Fig. 5C, D and Additional file 1: Fig. S2). These findings suggested that ANXA6 overexpression promoted autophagy in HNSCC cells. The AKT/mTOR signaling pathway is involved in tumor metastasis and is closely associated with autophagy [22, 33]. We found that ANXA6 overexpression inhibited the phosphorylation of AKT and mTOR (Fig. 5E), indicating a negative regulatory relationship between p-mTOR and autophagy in HNSCC. Based on these findings, ANXA6 inhibited mTOR phosphorylation, thereby promoting autophagy in HNSCC. To explore the effect of autophagy on HNSCC metastasis, we found that the invasion and migration abilities caused by ANXA6 overexpression were significantly inhibited after the addition of CQ (Fig. 6A, B). Finally, using human HNSCC samples, we found that autophagy was induced in HNSCC patients with LM (Fig. 6C). In summary, ANXA6 promotes the metastasis and invasion of HNSCC by inducing autophagy via inhibition of the AKT/mTOR pathway.

### ANXA6 promotes autophagy and metastatic ability in HNSCC cells by regulating TRPV2

**Fig. 7:**
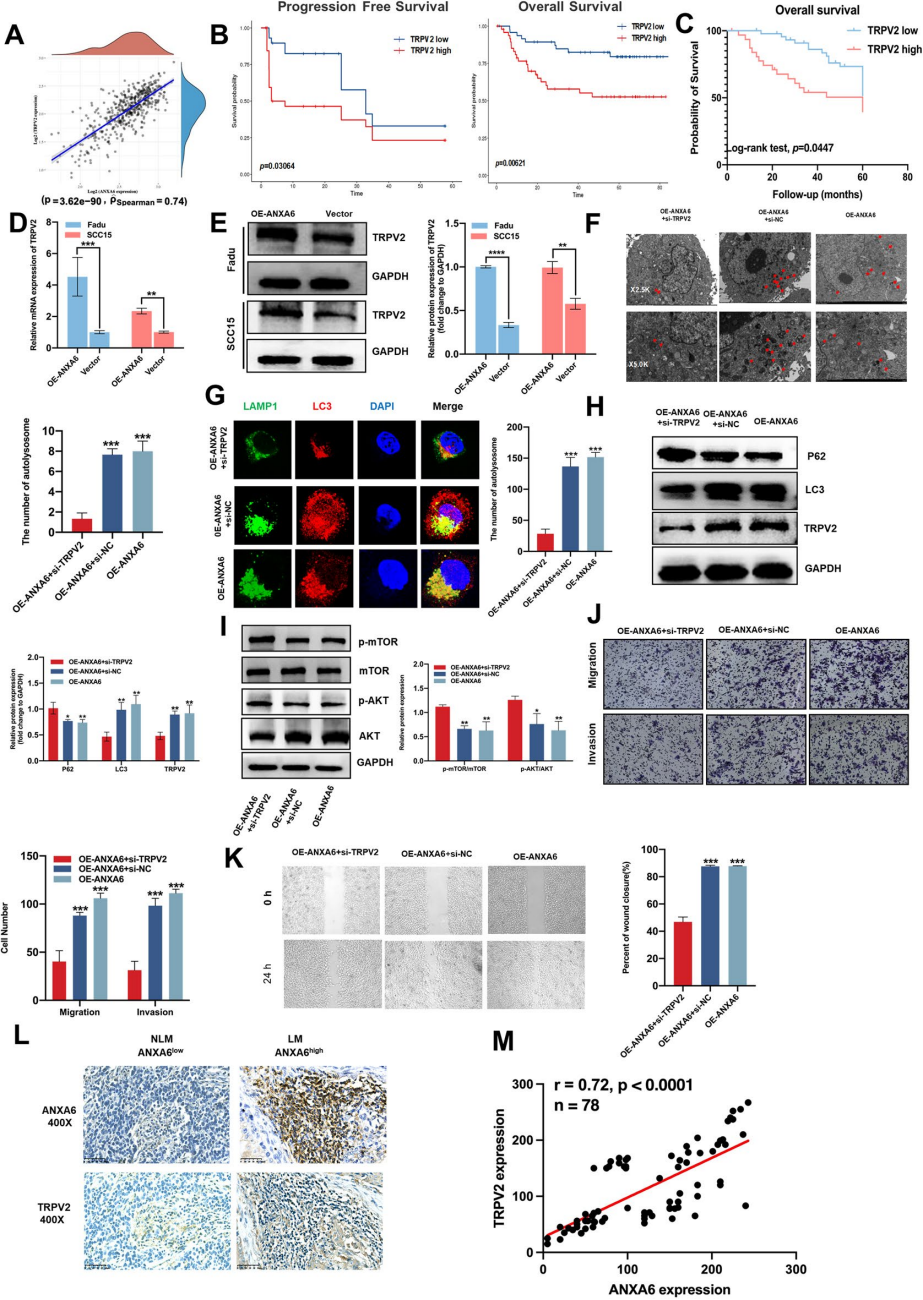
ANXA6 promotes autophagy and metastatic ability in HNSCC cells by regulating TRPV2. **A** Correlation between TRPV2 and ANXA6 according to the TCGA database of HNSCC. **B** Comparisons of overall survival between low TRPV2 expression group and high TRPV2 expression group in HNSCC patients using GEO database (GSE31056 and GSE27020). **C** The association between TRPV2 expression and mortality in HNSCC patients. **D** Relative mRNA expression levels of TRPV2 in HNSCC cells after overexpression ANXA6. **E** The protein expression levels of TRPV2 and ANXA6 in HNSCC cells after overexpression of ANXA6. **F** Representative transmission electron microscope (TEM) images of autophagosomes in ANXA6-overexpressed FaDu cell line and treated with siTRPV2. Magnification: ×25,000 for low-power fields, ×5000 for high-power fields. **G** Representative immunofluorescent images of the colocalization about LC3 (red) and LAMP1 (green) in ANXA6-overexpressed FaDu cell line and treated with siTRPV2. **H** The protein expression levels of P62, LC3, and TRPV2 in the ANXA6-overexpressed FaDu cell line treated with siTRPV2. **I** The protein expression levels of p-AKT, AKT, p-mTOR, and mTOR in ANXA6-overexpressed FaDu cell line and treated with siTRPV2. **J**, **K** Transwell assay (**J**) and wound healing assay (**K**) were used to assess the migration and invasion abilities in the ANXA6-overexpressed FaDu cell line and treated with siTRPV2. **L** The expression intensity of TRPV2 in HNSCC with lymphatic metastasis in vivo. M. The correlation analysis for expression intensity of TRPV2 and ANXA6 in 78 HNSCC clinical specimens. Data are presented as mean ± SD. *p < 0.05, **p < 0.01, ***p < 0.001, ****p < 0.0001; compared with the indicated group

To shed light on the signaling pathways involved in ANXA6-mediated autophagy. Using TCGA database, we analyzed the genes associated with ANXA6 expression to determine the specific mechanism by which autophagy induces LM in HNSCC. The targeted gene TRPV2 was ruled out because it can simultaneously affect tumor metastasis and autophagy (Fig. 7A) [34, 35]. A data mining study also revealed that high TRPV2 expression was associated with worse prognosis in patients with HNSCC using the Gene Expression Omnibus database (GSE31056 and GSE27020) (Fig. 7B). Interestingly, the Kaplan-Meier curve indicated that high TRPV2 expression also predicted poor outcomes of HNSCC in 78 HNSCC clinical specimens at follow-up (Fig. 7C). Next, both the mRNA and protein levels of TRPV2 were upregulated in response to ANXA6 overexpression in vitro (Fig. 7D, E). In ANXA6-overexpressed FaDu cells, TRPV2 KD reduced the formation of autophagosomes and autophagic lysosomes (Fig. 7F–H). Accordingly, p-AKT and p-mTOR protein levels were reversed (Fig. 7I). More importantly, TRPV2 KD attenuated tumor invasion and migration (Fig. 7J, K). Additionally, the effect of TRPV2 on metastasis was validated in the in vivo experiment. The expression levels of TRPV2 in HNSCC with LM were significantly higher than those in HNSCC without LM in 78 histochemical samples (Fig. 7L). Surprisingly, the expression levels of TRPV2 and ANXA6 were highly correlated (Fig. 7M). Taken together, these findings suggest that ANXA6 regulates TRPV2 by controlling AKT/mTOR signaling pathway-induced autophagy, thereby promoting LM in HNSCC.

## Discussion

**Fig. 8:**
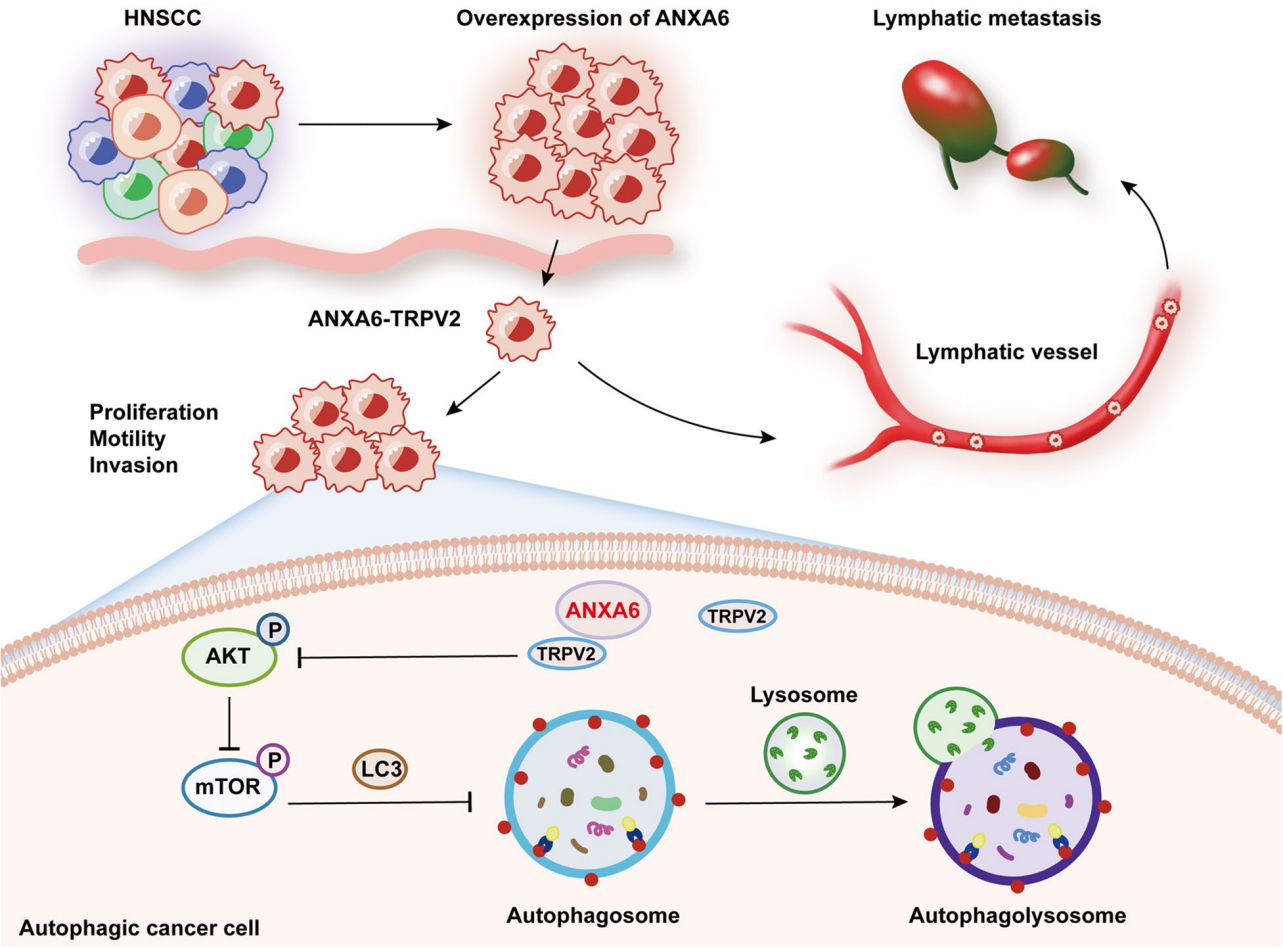
A schematic illustrating the proposed function of ANXA6 in regulation autophagy in HNSCC. ANXA6 was highly expressed in tumors from patients with HNSCC. By interacting with TRVP2, ANXA6 facilitates AKT/mTOR-induced autophagy, promoting proliferation, migration, and invasion of HNSCC cells, and eventually leads to lymphatic metastasis in HNSCC

HNSCC is the most common head and neck malignant tumor, occurring primarily in the mouth, pharynx, and larynx [1, 4]. Although HNSCC can be surgically resected and treated with radiotherapy, chemotherapy, and immunotherapy, only 30% of patients survive for 5 years after initial treatment [36]. LM is an important factor that affects the prognosis of HNSCC, and HNSCC patients with LM have an extremely lower survival [37]. LM in HNSCC is mainly caused by tumor proliferation, invasion, and migration. In the present study, we investigated ANXA6 and its regulatory mechanism affecting LM in HNSCC to provide a basis for targeted therapy for the disease (Fig. 8).

ANXA6 was identified as a possible prognostic marker for LM in HNSCC using transcriptome sequencing. ANXA6 is highly expressed in HNSCC tissues with LM and is associated with pathological N stages and poor overall survival rates. ANXA6 is a calcium-dependent membrane-binding protein with eight conserved sequences of 70 amino acids that form four repetitive modules [38, 39]. Moreover, it has been shown to affect tumor proliferation, invasion, migration, and apoptosis [40–42]. Previous studies have indicated that in certain tumor types and stages, ANXA6 may either suppress or promote tumorigenesis owing to its diverse functions. The current study demonstrated that ANXA6 KD in HNSCC cells can inhibit proliferation, invasion, and migration, and promote apoptosis, whereas ANXA6 overexpression can promote proliferation, invasion, and migration, and inhibit apoptosis of HNSCC cells. In an animal study, ANXA6 KD inhibited tumor proliferation and lymphatic dissemination. In conclusion, this is the first study to identify ANXA6 as a critical molecule that promotes LM in HNSCC.

As a scaffold protein, the most important roles of ANXA6 are signaling protein recruitment and membrane transport modulation, which affect cell motility via the Ras, Ras/MAPK, and/or FAK/PI3K signaling pathways in a variety of tumors [43]. However, the specific mechanism by which ANXA6 regulates LM in HNSCC is unknown. Autophagy may influence the occurrence and metastasis of tumors and regulate metabolism and homeostasis in the body; it has been reported that ANXA6 induces autophagy in cervical cancer [22]. However, no studies have examined whether ANXA6 affects autophagy during LM in HNSCC. GSEA conducted on the sequencing results of HNSCC clinical specimens showed that autophagy affected differentially enriched genes in HNSCC with LM. In thyroid and prostate cancer studies, autophagy was shown to promote tumor metastasis through Wnt/β-catenin and AMPK/mTOR signaling pathways [44, 45]. As we examined the protein markers of the AKT/mTOR pathway, we found that ANXA6 overexpression suppressed AKT and mTOR phosphorylation, which is consistent with mTOR-induced autophagy reported in a previous study [33]. These findings suggest that ANXA6 promotes LM in HNSCC by inhibiting mTOR phosphorylation and inducing autophagy.

To further investigate the regulatory mechanism between ANXA6 and autophagy, we analyzed the molecules affecting the association of ANXA6 in TCGA database and identified TRPV2 as a direct target of ANXA6. A positive regulatory relationship between ANXA6 and TRPV2 was demonstrated for the first time. TRPV2, one of six TRPV ion channel proteins, is a non-selective ion channel that is regulated by heat, mechanical stress, growth factors, and regulating intracellular Ca^2+^ concentration, plays an irreplaceable role in several physiological functions as well as in tumorigenesis and metastasis [24, 46]. Nabissi et al. showed that TRPV2 induces and activates autophagy to promote tumor progression [35], whereas Li et al. found that TRPV2 promotes esophageal squamous cell carcinoma by activating the HSP70/27 and PI3K/Akt/mTOR pathways [26]. However, few studies have examined the role of TRPV2 in LM. In the present study, we identified TRPV2 as a potential biomarker for HNSCC through data mining and in vivo and in vitro experiments. Additionally, TRPV2 KD reversed ANXA6-induced autophagy, HNSCC metastasis, and mTOR pathway protein changes. Collectively, these findings suggest that ANXA6 directly regulates TRPV2 and promotes autophagy by inhibiting mTOR phosphorylation, thereby promoting LM in HNSCC.

## Conclusion

This study demonstrates for the first time that ANXA6 promotes autophagy and LM in HNSCC by inhibiting mTOR phosphorylation, which regulates TRPV2 expression. These findings provide a theoretical basis for investigating the ANXA6/TRPV2 axis as a potential target for treating HNSCC and as a biomarker for predicting LM in HNSCC.

## Supplementary Information

**Additional file 1: Figure S1.** HNSCC stable cell lines construction. **Figure S2.** The quantitative statistical data of the Fig. 5C. **Table S1.** The primer sequences for qRT-PCR and siRNA target sequences. **Table S2.** The primary antibodies used in the study.

## Data Availability

All data from this study were used for the publication of this article and are guaranteed for availability.

## Abbreviations

HNSCC: Head and neck squamous cell carcinoma
ANXA6: Annexin A6
TCGA: The Cancer Genome Atlas
TRP: Transient receptor potential
LM: Lymphatic metastasis
NLM: Non-lymphatic metastasis
NC: Negative control
NAT: Normal adjacent tissues
shRNA: Short hairpin RNA
GSEA: Gene Set Enrichment Analysis
CQ: Chloroquine
