# Supplementary material for: ANXA6/TRPV2 axis promotes lymphatic metastasis in head and neck squamous cell carcinoma by inducing autophagy
Source: Exp Hematol Oncol. 2023 May 3;12:43. doi: 10.1186/s40164-023-00406-1 (PMC10155388; doi:10.1186/s40164-023-00406-1)
Supplement: Supplementary file 1 — Additional file 1: Figure S1. HNSCC stable cell lines construction. Figure S2. The quantitative statistical data of the Fig. 5C. Table S1. The primer sequences for qRT-PCR and siRNA target sequences. Table S2. The primary antibodies used in the study. [file 40164_2023_406_MOESM1_ESM.pdf]

Additional file 1

Figure S1

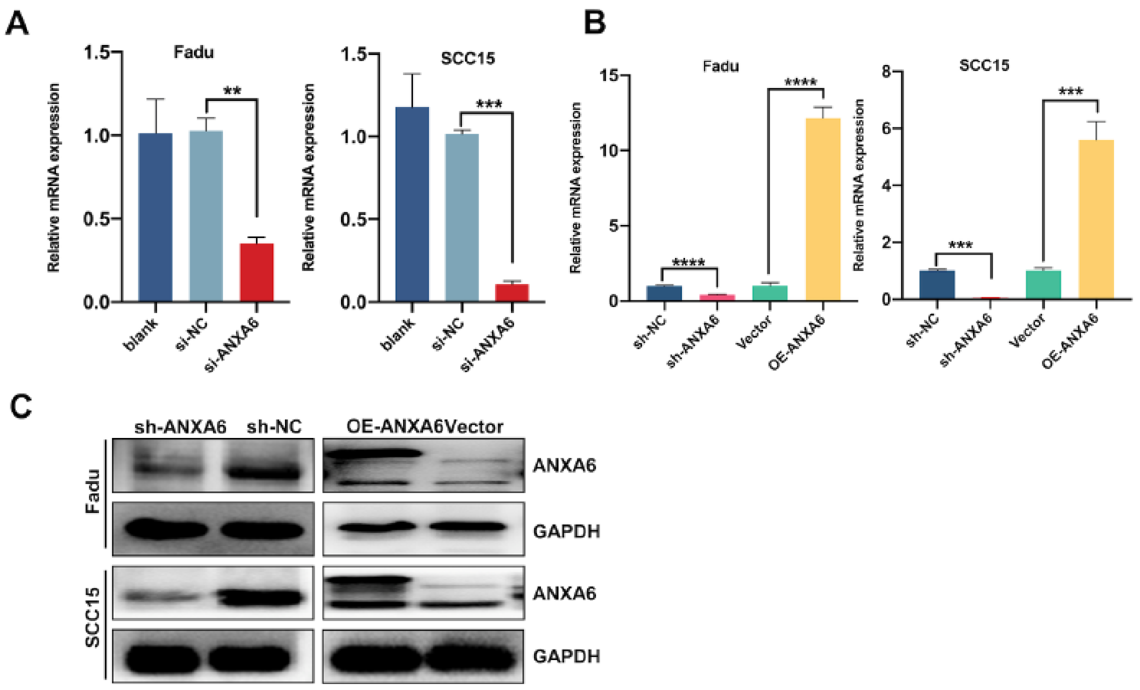

Fig. S1. HNSCC stable cell lines construction. **A**. Relative mRNA expression levels of ANXA6 in Fadu and SCC15 cell lines treated with siANXA6. **B.** Relative mRNA expression levels of ANXA6 in Fadu and SCC15 cell lines treated with lentivirus knockdown(sh-ANXA6) or overexpression of ANXA6(ANXA6). **C**. The protein expression levels of ANXA6 in Fadu and SCC15 cell lines treated with lentivirus knockdown or overexpression of ANXA6. Data are presented as mean±SD.

** $p < 0.01$, *** $p < 0.001$, ****$p < 0.0001$; compared with the indicated group.

Figure S2

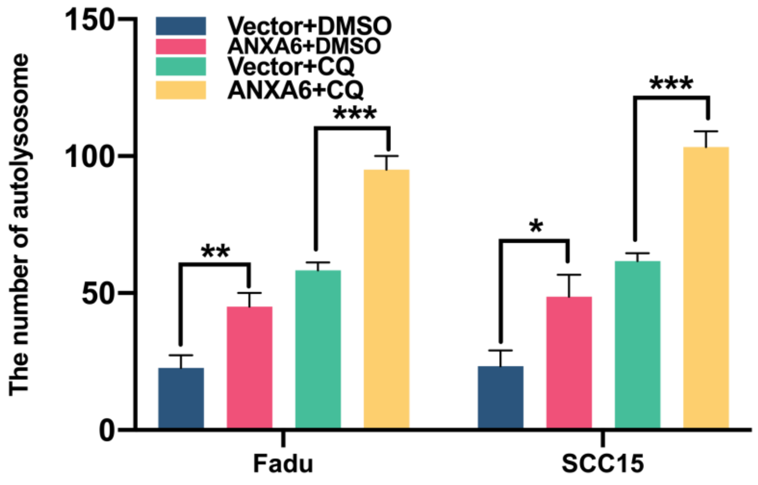

Fig. S2. The quantitative statistical data of the Figure 5C about thecolocalization of LC3 (red) and LAMP1 (green) after overexpression ANXA6 in HNSCC cells treated with DMSO (10μm/ml) or CQ (10μm/ml) for 24h. Data are presented as mean±SD. *p < 0.05, ** p < 0.01, *** p < 0.001; compared with the indicated group.

Table S1. Primer sequences for qRT-PCR and siRNA target sequences

Name	Forward (5'-3')	Reverse (3'-5')
ANXA6	ACGGTTGATTGTGGGCCTG	GTGCATCTGCTCATTGGTCC
GAPDH	CAGCGACACCCACTCCTC	TGAGGTCCACCACCCTGT
siANXA6	GGGACUUUGAGAAGCUAAUTT	AUUAGCUUCUCAAAGUCCCTT
TRPV2	TGGAGATCATTGCCTTTCATTGC	TTCTTCAGGGTAGGCTGATGGTA
siTRPV2	CCUAGUGAUGAUCUCGGACAA TT	UUGUCCGAGAUCAUCACUAGGTT

Table S2. The antibodies used in the current study			
**Name**	**Source**	**Identifier**	**Application**
ANXA6	Abcam	ab201024	WB: 1:1000
			IHC-P: 1:500
LC3B	Abcam	ab192890	WB: 1:2000
			IF: 1:500
LAMP1	Cell Signaling Technology	#15665T	IF: 1:100
SQSTM1/p62	Abcam	Ab207305	WB: 1:1000
Beclin1	Abcam	Ab207612	WB: 1:2000
GAPDH	Beyotime	AF1186	WB: 1:3000
TRPV2	ABclonal	A12367	WB: 1:1000
TRPV2	Abcam	Ab236955	IHC-P: 1:200
p-mTOR (Ser2448)	Cell Signaling Technology	#5536	WB: 1:1000
mTOR	Cell Signaling Technology	#2972	WB: 1:1000
p-AKT (phosho S129)	Abcam	Ab133458	WB: 1:1000
AKT	Cell Signaling Technology	#9272	WB: 1:1000
HRP-labeled Goat anti-rabbit IgG	Beyotime	A0208	WB: 1:5000
Goat-Anti-Rabbit IgG	Abcam	Ab150078	IF: 1:250
Anti-mouse IgG	Cell Signaling Technology	#4410	IF: 1:250
